# Effects of reducing inclusion rate of roughages by changing roughage sources and concentrate types on intake, growth, rumen fermentation characteristics, and blood parameters of Hanwoo growing cattle (*Bos Taurus coreanae*)

**DOI:** 10.5713/ajas.19.0269

**Published:** 2019-08-26

**Authors:** Seoyoung Jeon, Sinyong Jeong, Mingyung Lee, Jakyeom Seo, Dong Keun Kam, Jeong Hoon Kim, Jaehwa Park, Seongwon Seo

**Affiliations:** 1 Division of Animal and Dairy Sciences, Chungnam National University, Daejeon 34134, Korea; 2 Life and Industry Convergence Research Institute, Department of Animal Science, Pusan National University, Miryang 50463, Korea; 3 Cargill Agri Purina Inc., Seongnam 13630, Korea; 4 Department of Computer Science and Engineering, Chung-Ang University, Seoul 06974, Korea

**Keywords:** Blood Metabolites, Concentrate Type, Growing Korean Native Cattle (Hanwoo), Roughage Inclusion Rate, Roughage Source, Rumen Parameters

## Abstract

**Objective:**

Reducing roughage feeding without negatively affecting rumen health is of interest in ruminant nutrition. We investigated the effects of roughage sources and concentrate types on growth performance, ruminal fermentation, and blood metabolite levels in growing cattle.

**Methods:**

In this 24-week trial, 24 Hanwoo cattle (224±24.7 kg) were fed similar nitrous and energy levels of total mixed ration formulated using two kinds of roughage (timothy hay and ryegrass straw) and two types of concentrate mixes (high starch [HS] and high fiber [HF]). The treatments were arranged in a 2×2 factorial, consisting of 32% timothy–68% HS, 24% timothy–76% HF, 24% ryegrass–76% HS, and 17% ryegrass–83% HF. Daily feed intakes were measured. Every four weeks, blood were sampled, and body weight was measured before morning feeding. Every eight weeks, rumen fluid was collected using a stomach tube over five consecutive days.

**Results:**

The mean dry matter intake (7.33 kg) and average daily gain (1,033 g) did not differ among treatments. However, significant interactions between roughage source and concentrate type were observed for the rumen and blood parameters (p<0.05). Total volatile fatty acid concentration was highest (p<0.05) in timothy–HF-fed calves. With ryegrass as the roughage source, decreasing the roughage inclusion rate increased the molar proportion of propionate and decreased the acetate-to-propionate ratio; the opposite was observed with timothy as the roughage source. Similarly, the effects of concentrate types on plasma total protein, alanine transaminase, Ca, inorganic P, total cholesterol, triglycerides, and creatinine concentrations differed with roughage source (p<0.05).

**Conclusion:**

Decreasing the dietary roughage inclusion rate by replacing forage neutral detergent fiber with that from non-roughage fiber source might be a feasible feeding practice in growing cattle. A combination of low-quality roughage with a high fiber concentrate might be economically beneficial.

## INTRODUCTION

The consumption of a sufficient amount of roughage is critical for the development and maintenance of rumen health in cattle. An inadequate supply of roughage for cattle can cause short-term metabolic disorders, such as bloat and acidosis and can reduce volatile fatty acid (VFA) absorption in the long term [1]. In particular, rumen health problems during the growing stage can lead to negative effects, such as reduced feed intake and weight gain at the fattening (a.k.a. finishing) stage, therefore, it is recommended that enough high quality roughage is provided for growing cattle [2].

In some areas where there is a shortage of pastures, farmers rely mostly on imported roughage and supplying a large amount of roughage is quite challenging. Especially in Korea and Japan, where beef cattle are raised for a long period (e.g., 30 months of age), providing sufficient amounts of roughage during the growing period is even more important. In these countries, the expense of high quality imported roughage is a burden on producers [3]. Thus, finding ways to reduce roughage feeding or finding cheaper feed ingredients to replace roughage without negatively affecting rumen health has been of interest in these countries.

One way to reduce the amount of roughage in the diet is to use relatively low-quality roughage. Since low quality roughage (e.g., straws and stalks) has a higher fiber content per unit mass than high quality roughage, a smaller amount of low quality roughage can meet the fiber requirement of animals. Galyean and Defoor [4] analyzed the literature for the effect of roughage source and level on the intake of feedlot cattle, and concluded that not the amount of roughage itself, but the amount of neutral detergent fiber (NDF) supplied by roughage, was the most important factor determining the intake of feedlot cattle. It has also been reported that the level of NDF supplied by roughage as well as non-fiber carbohydrate (NFC) is more important than the source of the roughage for rumen function and the performance of dairy cows [5,6].

The non-roughage fiber sources (NRFS) contained in the concentrate mix can replace some roughage fiber. The NRFS, such as soybean hulls, beet pulp, rice bran, and other fibrous agricultural byproducts, are relatively inexpensive and contain more digestible fiber, protein, and minerals than high quality roughage. Hsu et al [7] reported the possible use of NRFS from fibrous agricultural byproducts as alternatives to roughage sources for calves. Weidner and Grant [8] reported that high quality roughage can be replaced by soybean hulls and coarsely chopped hay in lactating dairy cows. In their study, the addition of coarsely chopped hay along with soybean hulls increased the rumen mat consistency and ruminal mean retention time (RMRT) of particles. The replacement of roughage NDF from corn silage with NRFS NDF from soybean hulls in total mixed ration (TMR) increased the milk fat yield (kg/d) without compromising the intake or animal health of dairy cows [9]. The same research group also showed increases in dry matter intake (DMI) and milk production when NDF supplied by roughage was replaced by NDF supplied by soybean hulls [10]. Most of the studies on replacing roughage fiber with NRFS or the effects of roughage source on animal performance have been carried out in dairy cows and finishing cattle, and there are few studies on reducing roughage sources for growing cattle.

Therefore, we conducted this study to investigate the effects of roughage inclusion levels by altering roughage sources and concentrate types given to growing cattle. The experiment was conducted with a 2×2 factorial design with two kinds of roughage and two types of concentrate mix, providing the similar level of energy and protein in the diet. Analyses were conducted for growth performance, ruminal fermentation, and blood metabolites.

## MATERIALS AND METHODS

This study was conducted at Center for Animal Science Research, Chungnam National University, Korea. The animal use and protocols for this experiment were reviewed and approved by the Chungnam National University Animal Research Ethics Committee (CNU-00715). The experimental design and statistical analyses performed in this study were consistent with recently published guidelines for statistical analyses in animal studies [11].

### Animals, housing, and dietary treatments

A total of 24 Hanwoo growing cattle (body weight [BW], 224± 24.7 kg; 7 months old) participated in this six-month (24 weeks) feeding trial. One male and two female cattle with similar BWs were grouped and housed in a pen. Each pen (5 m×5 m) was equipped with one feed bin which allowed us to measure individual feed intake automatically by recognizing each animal using the radio-frequency identification tag attached to them (Dawoon Co, Incheon, Korea).

Two sources of roughage (timothy hay and ryegrass straw) and two types of concentrate mix (high starch [HS] and high fiber [HF]) were prepared to formulate four TMRs containing similar levels of energy and protein to meet the nutrient requirements to support an average daily gain (ADG) of 850 g/d [12]. The timothy and ryegrass contained 71 g/kg crude protein (CP), 649 g/kg NDF, and 533 g/kg total digestible nutrient (TDN), and 44 g/kg CP, 751 g/kg NDF, and 507 g/kg TDN on dry matter (DM) basis, respectively. Timothy had a higher quality than ryegrass; therefore, the highest roughage inclusion was possible when timothy was used as the forage source. The feed composition of the concentrate mixes and the analyzed chemical composition of the experimental TMRs are given in Tables 1 and 2. Each group of cattle was randomly allocated to one of the four TMR treatments arranged as a 2×2 factorial: i) 32% timothy and 68% HS (32% roughage; THS), ii) 24% timothy with 76% HF (24% roughage; THF), iii) 24% ryegrass with 76% HS (24% roughage; RHS), and iv) 17% ryegrass with 83% HF (17% roughage; RHF). The cattle were fed twice daily at 0900 and 1800 h. The TMR, drinking water, and mineral blocks were freely accessible to the animals throughout the experiment.

### Measurements and sample collection

Daily feed intakes were averaged over 4 weeks. When averaging, daily intakes that were less than 1 kg, greater than 2.5 times the standard deviation (SD), or smaller than 2.5 times the SD were treated as outliers and omitted. The BW was measured every 4 weeks before morning feeding.

The TMRs were sampled regularly and stored at −20°C until subsequent chemical analysis. Every 8 weeks, rumen fluid was collected over five consecutive days at a different time each day (0800, 1100, 1300, 1500, and 1700) from one group of cattle from each treatment (12 cattle in total) which were priori and randomly selected. Rumen samples were collected using an oral stomach tube 2 h after each morning feeding as described by Lee et al [13]. Briefly, after discarding the initially obtained rumen fluid (approximately 200 mL), 500 mL of rumen fluid was collected in a glass flask. The pH of the rumen fluid was analyzed immediately after its collection using a general-purpose pH meter (EcoMet P25, Istek, Inc., Seoul, Korea), and then the rumen fluid was transferred to the laboratory. After centrifugation of the rumen fluid at 21,000×*g* for 10 min at 4°C, the supernatant was frozen at −20°C until the analyses for VFAs and ammonia nitrogen concentration.

Blood samples were taken every 4 weeks before measuring the BW from another group of cattle from each treatment (12 cattle in total), which was different to the group used for collecting rumen fluid. Approximately 20 mL of blood was taken from the jugular vein of each calf and collected into a vacutainer tube clot activator (BD Vacutainer; BD and Co., Franklin Lakes, NJ, USA). Serum was obtained by centrifugation at 1,300×*g* for 15 min at 4°C and frozen at −80°C until later analysis.

### Chemical analysis

The diet samples were dried at 60°C for 96 h and ground through a cyclone mill (Foss, Hillerød, Denmark) fitted with a 1 mm screen before chemical analysis. The nutrient composition of the samples was analyzed at Cumberland Valley Analytical Services Inc. (Hagerstown, MD, USA). References of the analytical methods used for chemical analysis in this study are fully described elsewhere [14]. The content of DM (#934.15), CP (#990.03), ether extract (#920.39), acid detergent fiber (#973.18), and ash (#942.05) were determined. CP was calculated as 6.25 times the nitrogen content, and the total nitrogen was measured using the Dumas method using a Leco FP-528 Nitrogen Combustion Analyzer (Leco Inc., Saint Joseph, MI, USA). The acid detergent lignin (ADL) content was analyzed and the neutral detergent fiber (aNDF) content was analyzed using a heat stable amylase and expressed inclusive of residual ash. The soluble protein, neutral detergent insoluble crude protein (NDICP) and acid detergent insoluble crude protein (ADICP) contents were also determined. The contents of ethanol soluble carbohydrate (ESC), starch, and macro- and micro-mineral content were determined.

The content of TDN, net energy for maintenance, and net energy for growth were estimated using National Research Council [12] equations. The dietary carbohydrate and protein fractions were estimated according to the Cornell Net Carbohydrate and Protein System [15] with the following modifications. For the carbohydrate fractions, carbohydrate A fraction (CA) was sugars and organic acids assumed to be equal to ESC, carbohydrate B1 fraction (CB1) was starch, CB2 was soluble fiber calculated as NFC–CA–CB1, CB3 was available NDF estimated by aNDF–NDICP minus 2.4 times ADL, and carbohydrate C fraction (CC) was unavailable carbohydrate estimated by 2.4 times ADL. In the protein fractions, PA+B1 was soluble protein which was equal to soluble CP (SOLP), PB2 (Protein B2 fraction) was intermediate degradable CP estimated by 100–NDICP–SOLP, protein B3 fraction (PB3) was slowly degradable fiber-bound CP estimated by NDICP–ADICP, and protein C fraction (PC) was unavailable CP which was equal to ADICP. All the carbohydrate and protein fractions were expressed as g/kg of total carbohydrate or CP, respectively.

The NH_3_-N concentration of the rumen fluid was determined as follows. Following re-centrifugation of the rumen fluid at 21,000×*g* for 15 min, 20 μL of the supernatant was mixed with 1 mL of phenol color reagent and 1 mL of alkali-hypochlorite reagent. The mixture was then incubated in a water bath for 15 min at 37°C. After being mixed with 8 mL of distilled water, the optical density of the mixture was measured at 630 nm, using a spectrophotometer (UV-1800, Shimadzu, Kyoto, Japan).

In order to determine the VFA concentration of rumen fluid, rumen fluid supernatant (1 mL) was mixed with 0.2 mL of metaphosphoric acid (250 g/L) and kept at 4°C for 30 min. Following centrifugation of the mixture at 21,000×*g* for 10 min at 20°C, the supernatant was injected into a gas chromatograph (HP 6890, Hewlett-Packard Co., Palo Alto, CA, USA) equipped with a flame ionization detector and capillary column (Nukol Fused silica capillary column 30 m× 0.25 mm× 0.2 μm, Supelco Inc., Bellefonte, PA, USA). The temperature of the oven, injector, and detector was 90°C to 180°C, 185°C, and 210°C, respectively. Nitrogen was used as the carrier gas at a flow rate of 40 mL/min.

The serum was analyzed for total protein (TP), aspartate transaminase (AST), alanine transaminase (ALT), glucose, total cholesterol, triglycerides, non-esterified fatty acid (NEFA), blood urea nitrogen (BUN), creatinine, calcium (Ca), inorganic phosphate (IP), magnesium (Mg), and albumin using kits purchased from Wako Pure Chemical Industries, Ltd. (Osaka, Japan) and a clinical auto analyzer (Toshiba Accute Biochemical Analyzer-TBA-40FR, Toshiba Medical Instruments, Tokyo, Japan).

### Statistical analysis

Data were analyzed using PROC MIXED (SAS institute, Cary, NC, USA). The linear model was as follows:

$$y_{ij}=\mu +\tau_{i}+e_{ij}$$

where, *y_ij_* is the jth observation (j = 1–24) in the ith treatment (i = 1–4), *μ* is the overall mean, *τ_i_* is the fixed effect of the ith treatment, and *e_ij_* is the unexplained random effect on the jth observation in the ith treatment.

Three orthogonal contrasts were tested for the difference between two roughage sources (timothy hay vs ryegrass straw), between two concentrate types (high starch vs high fiber), and the interaction between roughage source and concentrate type. Individual means were also compared by a Tukey’s test. To test the effects of treatments on rumen and blood parameters, the data were analyzed as repeated measures to account for the correlation between repeated measurements of each animal. For this analysis, no structure was assumed for the variance-covariance matrix. Statistical significance was declared at p<0.05, and a trend was discussed at 0.05≤p<0.1.

## RESULTS AND DISCUSSION

With the aim to reduce the roughage content of the diet of growing cattle without negative effects on rumen health, this study investigated the effect of different roughage sources and concentrate types on the growth performance, rumen fermentation, and blood profiles of Hanwoo growing cattle. So far, most studies on replacing roughage fiber with NRFS or the effects of roughage source on animal performance have been carried out in dairy cows and finishing cattle, and there have been few studies on growing beef cattle, especially when the level of roughage inclusion in the diet was less than 50%. In the present study, the highest roughage inclusion (i.e., 32%) and the lowest roughage inclusion (i.e., 17%) were possible when timothy and ryegrass, respectively, were used as the roughage source. Although both the timothy and ryegrass used in this study were graded as low quality roughage based on the criteria (i.e., CP<8%, TDN<55%) proposed by Leng [16], timothy had a higher quality and is more expensive than ryegrass. The market price of timothy was 2.25 times higher than that of ryegrass (459 vs 203 KRW/kg) in 2014.

### Growth performance

The source of dietary NDF can affect the growth performance of calves. In this study, however, the substitution of roughage with NRFS by changing roughage sources and concentrate types did not affect growth performance, including DMI, ADG, and the feed conversion ratio (the DMI in kg to ADG in kg ratio) when the energy and protein content was similar (p>0.05, Table 3). Low quality roughage, which contains a large amount of NDF, can reduce feed intake because NDF induces satiety due to physical fill and a long RMRT [17]. A longer RMRT, however, can also increase roughage digestibility, which can lead to the enhanced growth of animals [18]. NRFS has a small particle size and high specific gravity [19], and thus its ruminal passage rate is high, which might increase feed consumption due to a lower physical fill. Adin et al [10] reported an increased feed intake when corn silage and vetch hay were partially replaced by soybean hull and wheat bran. Miron et al [9], however, reported that there was no difference in TMR intake when some of the corn silage was replaced with soybean hulls.

The amount of NDF, regardless of its origin, is known to affect feed intake and thus the growth performance of cattle. When the dietary NDF content was the same, the roughage source did not affect ADG or growth efficiency [18]. A similar finding was also reported by Eastridge et al [5]. In addition, there was no difference in DMI and milk production when alfalfa was replaced by a smaller amount of rice straw maintaining the same level of NDF in lactating dairy cows [6]. In the present study, the NDF content was similar among the TMRs, which might have resulted in no difference in the feed intake or growth performance among the treatments.

### Rumen fermentation

The ruminal pH differed between the roughage sources (p = 0.05, Table 4). The cattle fed timothy had a lower ruminal pH (6.42) than those fed ryegrass (6.47), mainly due to the low pH of the THF group fed timothy with a lower roughage inclusion rate (THF, 24%; 6.33). A low pH indicates that a large amount of organic acid has been produced in the rumen, and similarly, a higher ruminal total VFA concentration was observed in the THF group than the other treatment groups in the present study. This result, however, was not consistent with the study conducted Adin et al [10]. Adin et al [10] showed that ruminal pH numerically increased (6.67 vs 6.57) when roughage NDF was replaced by the NDF supplied by soy hulls. Their finding is surprising, since the experimental TMR in which roughage was replaced with soy hull had a lower roughage inclusion rate (23.5% vs 35.0%), roughage NDF content (12.8% vs 18.7%), and physical effective NDF (>8, 11.7% vs 14.1%) than the control TMR. Moreover, the experimental diet showed a long RMRT of the roughage particles despite a higher DMI, which conflicted with the general observation of a negative correlation between RMRT and DMI [20].

The total VFA concentration in the rumen significantly differed between the dietary treatments (p<0.01, Table 4). The highest total VFA concentration was observed in the THF group, which was significantly higher than in the RHS group that was fed TMR with ryegrass and a high starch concentrate (p<0.05). More interestingly, a significant interaction between roughage source and the concentrate mix type was observed for the total VFA concentration and the acetate: propionate ratio (AP ratio). A lower inclusion of roughage increased the total VFA concentration more when timothy was fed (73.9 mM to 81.2 mM) than when ryegrass was fed (69.5 mM to 75.7 mM). When ryegrass was provided, a decrease in roughage inclusion resulted in a higher molar proportion of propionate and a lower AP ratio (p<0.001), which was as expected. The opposite trends, however, were observed when timothy was the roughage source.

As the ruminal fermentation of fiber carbohydrates is slower and produces more acetate, while that of NFC is faster and produces more propionate, a decrease in the roughage to concentrate ratio generally increases the total VFA concentration and decreases the AP ratio in the rumen. The results of the total VFA concentrations in the present study agree with this general observation. However, the significantly higher total VFA concentration in THF compared to RHS group was somewhat unexpected as their chemical composition and predicted digestibility were similar. Abdelhadi and Santini [21] reported that roughage source did not affect the total VFA concentration or relative molar proportions of short chain VFA. In addition, there was no difference in rumen parameters when alfalfa was replaced by a smaller amount of rice straw maintaining the same level of NDF in lactating dairy cows [6]. Conversely, Highfill et al [22] reported that the total VFA was higher in the citrus pulp supplemented group (fescue hay+citrus pulp+soy hull) than the corn supplemented group (fescue hay+corn+soy hull) after 2 and 4 hours of feeding; however, the NDF and CP content of the diets was the same. Unfortunately, they were not able to provide reasonable answers for what caused this difference. In the Poore et al [23] study, although there was no significant difference in the total VFA concentration, the AP ratio of the high quality forage (i.e., alfalfa) and low starch concentrate (i.e., dry rolled sorghum) group was higher than that of the low quality roughage (i.e., wheat straw) and high starch concentrate (i.e., steamed-flaked sorghum) group. These authors also could not find a reason for this phenomenon. Further research is needed to reveal the underlying mechanism for why the THF group had a higher total VFA concentration than the other groups with no change in the AP ratio.

No significant difference in the ruminal NH_3_-N concentration was observed among the treatments (p>0.05), which was consistent with other studies. The ammonia concentration in the rumen is related to the ruminal passage rate, which is mainly determined by DMI [20], and the ruminal nitrogen balance, which is determined by the dietary rumen degradable protein (RDP) content and microbial nitrogen uptake [24]. Javaid et al [25] reported that there was no difference in the NH_3_-N concentration of the rumen fluid if the RDP (PA+PB1+PB2) concentration of the experimental diets was similar. There was no difference in the ruminal NH_3_-N concentration when dairy cows were fed iso-nitrous and iso-NDF TMR, even though the roughage source and inclusion level were different [6]. Our present study also indicates that ruminal NH_3_-N concentrations might not be different if the dietary RDP and DMI are similar.

### Blood metabolites

Significant interactions between roughage source and the concentrate type were observed for most blood parameters (p< 0.05, Table 5). Significant differences among the treatments were observed for the concentrations of plasma TP, ALT, Ca, IP, total cholesterol, triglycerides, and creatinine (p<0.05), mainly owing to the interaction between roughage and the concentrate mix. Additionally, the contrast analysis identified significant interactions between roughage source and the concentrate mix for AST, BUN, and albumin concentrations (p< 0.05). The effect of the treatments on the plasma concentration of TP, AST, Ca, and albumin was similar; their concentration increased as the roughage inclusion decreased, but the extent of the increase was smaller in the cattle fed timothy than in those fed ryegrass. However, a decrease in the roughage content increased the BUN and IP concentrations, although the extent of the increase was greater in the cattle fed timothy than in those fed ryegrass. Interestingly, as the inclusion rate of roughage decreased, the concentration of triglyceride in the blood increased in the cattle fed timothy, whereas it decreased in those fed ryegrass. The concentration of triglyceride was the only metabolite that showed this pattern of interaction.

The plasma creatinine concentration was the only parameter that showed a significant difference among treatments differed by the type of concentrate mix. Compared to the HS group, the HF group had a lower blood creatinine concentration (p = 0.02). On the other hand, the roughage source, as well as its interaction with the concentrate mixes, altered the concentration of IP and cholesterol in the blood (p<0.05). The concentration of total cholesterol in the blood was higher in the cattle fed timothy than in those fed ryegrass (p = 0.008). Furthermore, the lower inclusion of roughage increased the cholesterol level more when timothy was fed than when ryegrass was fed (p<0.001).

Changes in the concentration of metabolites associated with protein metabolism appeared to be related to the level of dietary CP. In the present study, the CP content in the HF concentrate mix was unintentionally higher than that in the HS concentrate mix, which lead to a higher dietary CP with a higher inclusion of concentrate. The responses of serum TP, AST, ALT, BUN, albumin, and creatinine concentrations in the present study are consistent with general observations. The concentration of TP and albumin in the serum is correlated with the level of protein in the diet [26]. A decrease in serum creatinine concentration indicates catabolism of protein [27], and when tissue breaks down, AST and ALT concentrations also increase [28]. The BUN level increases with a high RDP content in feed, and rarely increases by recycled nitrogen when dietary nitrogen is depleted [29].

Significant changes in the concentration of cholesterol and triglycerides suggest that lipid metabolism in cattle can be altered by interactions between the sources of roughage and type of concentrate mix. The cattle fed timothy had a higher cholesterol level than those fed ryegrass. A lower inclusion of roughage increased serum cholesterol with both roughage sources. The lower inclusion of roughage, however, increased the triglyceride concentration in the cattle fed timothy, whereas it decreased the triglyceride concentration in those fed ryegrass. The serum cholesterol concentration, together with glucose, NEFA, and triglycerides, is a parameter that can indicate the state of energy metabolism. When nutritional supply is insufficient, the concentration of cholesterol decreases, and vice versa [30]. O’Kelly [31] reported that plasma cholesterol and triglyceride (TG) increased when high quality roughage (alfalfa hay) was fed. In the study by Campanile et al [32], the total cholesterol was higher in the high energy diet than the low energy diet, but there was no difference in the TG level.

Among the carbohydrate fractions, the CA and CB1 fractions are used rapidly in the rumen, whereas the CB2 and CB3 fractions are used more slowly in the rumen. During rumen fermentation, the CA and CB1 fractions are glucogenic and produce propionate, whereas the CB2 and CB3 fractions are lipogenic and produce acetate. In this study, THF was the most lipogenic and RHS was the most glucogenic of the carbohydrate fractions, even though the difference was small. According to Van Knegsel et al [33], the blood cholesterol level was higher in cattle fed a lipogenic diet than in those fed a glucogenic diet. In their study, the blood TG concentrations were not correlated with the lipogenicity of the diet, which is consistent with our results [34]. It is presumed that changes in the blood TG concentration are more dependent on the metabolic ability of the liver and the transport of TG than the lipogenicity of the diet.

Interactions between roughage source and concentrate type might also alter mineral metabolism in growing cattle. In the present study, the concentration of Ca and IP was affected by the interaction effect of roughage and concentrate (p<0.05). Minerals such as Ca, IP, and Mg play an important role in digestion and skeleton formation [26]. Ca is mostly used to construct body structures such as bones and teeth [26]. IP is involved in many metabolic reactions, such as energy transfer. Since there was no difference in the Ca, P, and Mg content among the diets and there was no difference in dietary intake, it is considered that the difference in blood macromineral level according to treatment is not due to the diet factor (i.e., dietary mineral composition and/or intake level) [35].

Overall, the significant interactions for the ruminal fermentation parameters and blood metabolites between roughage source and concentrates type indicate that the effect of the combination of roughage and concentrate needs to be considered in growing cattle. Although there was no difference in the growth performance in this study, differences in growth performance and/or carcass characteristics might be found when long-term testing is performed if there are differences in metabolism. Most studies on the effects of roughage source or replacing roughage fiber with NRFS on animal performance have been carried out on dairy cows and finishing cattle. This study is the first study to investigate the effects of roughage and fiber source on growth performance, rumen metabolism, and body metabolism of growing beef cattle. The results of this study suggest that the source of roughage and concentrate type might affect rumen metabolism and body metabolism in the short term, but does not affect the growth performance of the growing beef cattle. However, since the experiment was conducted over only 6 months of the growing period, the effects of roughage source and concentrate type on growth performance, and rumen and body metabolism are unknown in the long term. Therefore, future studies on the effects of roughage source and concentrate type on growth performance and carcass characteristics are needed. In addition, additional studies on rumen microbial composition and nutrigenomics are needed to elucidate the effects of NRFS and low-quality roughage on rumen fermentation and blood metabolites.

## IMPLICATIONS

We concluded that the rumen fermentation and body metabolism of the growing calves were affected by the roughage sources and concentrate types in the diet, even though growth performance did not significantly differ. Based on the results of growth performance, ruminal VFA concentrations, and the concentration of blood metabolites, it appears that a decrease in the inclusion rate of roughage in the diet by replacing NDF from forage with that from NRFS could be a feasible feeding practice in growing cattle. Considering the price of feed ingredients, a combination of a low-quality roughage with a high NRFS containing concentrate mix might be economically beneficial. Additional studies, however, are needed to elucidate the longer associative effects of roughage source and concentrate type on rumen and body metabolism and carcass characteristics in beef cattle.

## Figures and Tables

**Table 1:** Diet composition (g/kg) of experimental concentrate mixes

Items	High starch concentrates	High fiber concentrates
Corn	519.3	335.8
Wheat	55.5	55.4
Gluten feed	203.0	290.4
Rice bran	86.8	63.7
Soy hulls	22.3	144.1
Soybean meal	33.2	33.1
Molasses	27.0	26.9
Limestone	25.2	22.8
CMS/MSG1)	7.0	11.9
Urea	8.9	1.4
Salt	5.1	5.0
Ammonium chloride	4.8	7.9
Mineral premix2)	1.2	1.2
Vitamin premix3)	0.6	0.6

1) Condensed molasses solubles from the process of making monosodium glutamate

2) 667 mg/kg Co, 13,333 mg/kg Cu, 1,333 mg/kg I, 33,333 mg/kg Fe, 66,667 mg/kg Mn, 133 mg/kg Se, 100,000 mg/kg Zn.

3) 10,870,000 IU/kg vitamin A, 6,522,000 IU/kg vitamin D, 47,826 IU/kg vitamin E.

**Table 2:** Analyzed chemical composition (g/kg DM or as stated) of experimental total mixed ration

Items	Timothy1)		Ryegrass2)	
	
	High starch	High fiber	High starch	High fiber
DM (g/kg as fed)	886	885	872	876
OM	933	926	938	938
CP	131	142	134	145
SOLP	56	58	62	69
NDICP	12	18	13	16
ADICP	9	11	10	11
Dietary aNDF	388	401	374	395
Roughage aNDF	208	156	180	128
ADF	231	230	220	221
ADL	40	39	39	40
Ether extract	36	40	37	36
Ash	67	74	62	62
Ca	7	9	8	8
P	4	4	4	5
K	14	14	11	11
Na	2	3	3	3
Cl	8	9	8	8
S	3	3	3	3
Mg	2	3	2	3
TDN	699	695	710	703
NEm (MJ/kg DM)	6.7	6.6	6.9	6.8
NEg (MJ/kg DM)	4.2	4.1	4.3	4.2
Total carbohydrates	766	745	767	757
NFC	389	362	406	377
Carbohydrate fraction3) (g/kg carbohydrate)				
CA	84	80	69	58
CB1	331	302	378	358
CB2	94	105	83	83
CB3	368	387	348	374
CC	124	127	123	126
Protein fraction4) (g/kg CP)				
PA+B1	428	412	460	476
PB2	483	460	444	413
PB3	23	53	21	35
PC	67	75	75	76

DM, dry matter; OM, organic matter; CP, crude protein; SOLP, soluble CP; NDICP, neutral detergent insoluble CP; ADICP, acid detergent insoluble CP; aNDF, neutral detergent fiber analyzed using a heat stable amylase and expressed inclusive of residual ash; ADF, acid detergent fiber; ADL, acid detergent lignin; TDN, total digestible nutrients; NEm, net energy for maintenance; NEg, net energy for growth; NFC, non-fiber carbohydrate.

1) Contained 878 g/kg DM and 928 g/kg OM, 71 g/kg CP, 649 g/kg aNDF, 533 g/kg TDN, and 4.9 MJ/kg NEm on DM basis.

2) Contained 890 g/kg DM and 937 g/kg OM, 44 g/kg CP, 751 g/kg aNDF, 507 g/kg TDN, and 4.5 MJ/kg NEm on DM basis.

3) CA, carbohydrate A fraction, ethanol soluble carbohydrates; CB1, carbohydrate B1 fraction, starch; CB2, carbohydrate B2 fraction, soluble fiber; CB3, carbohydrate B3 fraction, available insoluble fiber; CC, carbohydrate C fraction, unavailable carbohydrate.

4) PA+B1 protein A and B1 fractions, soluble CP; PB2, protein B2 fraction, intermediate degradable CP; PB3, protein B3 fraction, slowly degradable fiber-bound CP; PC, protein C fraction, unavailable CP.

**Table 3:** Effect of the quality of forage and concentrate mix on growth performance and metabolic energy intake in Hanwoo calves (n = 24)

Items	Treatment1)				SEM	p-value			
	
	Timothy		Ryegrass			Overall	Contrast		
		
	HS	HF	HS	HF			Forage	Concentrate	Interaction
Initial BW (kg)	191	196	192	192	9.6	0.988	0.908	0.841	0.788
Final BW (kg)	351	369	354	351	8.7	0.788	0.594	0.621	0.483
ADG (g/d)	1,011	1,081	988	1,054	62.7	0.728	0.698	0.294	0.970
DMI (kg/d)	7.2	7.7	7.4	7.1	0.51	0.866	0.764	0.858	0.447
FCR	7.1	7.2	7.5	6.8	0.48	0.797	0.999	0.534	0.442
ME intake (MJ/d)	70.5	71.9	71.2	69.9	4.81	0.992	0.898	0.992	0.777

SEM, standard error of the mean; BW, body weight; BW, body weight; ADG, average daily gain; DMI, dry matter intake; FCR, feed conversion ratio; ME, metabolizable energy.

1) HS, high starch concentrate, HF, high fiber concentrate.

**Table 4:** Effect of the quality of forage and concentrate mix on rumen parameters in Hanwoo calves (n = 12)

Items	Treatment1)				SEM	p-value			
	
	Timothy		Ryegrass			Treatment	Contrast		
		
	HS	HF	HS	HF			Forage	Concentrate	Interaction
pH	6.50	6.33	6.52	6.41	0.060	0.050	0.025	0.103	0.315
NH_3_-N (mg/dL)	8.87	10.12	7.25	10.15	1.296	0.376	0.226	0.262	0.564
Total VFA2) (mM)	73.9^ab^	81.2^a^	69.5^b^	75.7^ab^	2.51	0.002	0.052	0.158	0.001
Acetate (mmol/mol)	640.1	645.3	650.3	648.5	3.24	0.221	0.141	0.185	0.430
Propionate (mmol/mol)	205.8^ab^	198.6^ab^	194.5^b^	210.4^a^	3.46	0.023	0.257	0.219	0.006
Butyrate (mmol/mol)	145.7	152.2	149.7	143.7	2.32	0.028	0.330	0.827	0.005
A:P ratio	3.15^ab^	3.24^ab^	3.40^a^	3.05^b^	0.060	0.007	0.176	0.033	0.003

SEM, standard error of the mean; VFA, volatile fatty acid; A:P ratio, ratio of acetate and propionate.

1) HS, high starch concentrate; HF, high fiber concentrate.

2) Total VFA, sum of acetate, propionate, isobutyrate, butyrate, isovalerate and valerate.

a,b Means that do not have common superscripts significantly differ within the treatments (p<0.05).

**Table 5:** Effect of the quality of forage and concentrate mix on blood metabolites in Hanwoo calves (n = 12)

Items	Treatment1)				SEM	p-value			
	
	Timothy		Ryegrass			Treatment	Contrast		
		
	HS	HF	HS	HF			Forage	Concentrate	Interaction
Total protein (g/dL)	3.25^ab^	3.57^a^	2.96^b^	3.42^ab^	0.132	0.027	0.101	0.644	0.008
AST (IU/L)	39.88	42.69	35.95	47.72	2.371	0.104	0.300	0.265	0.041
ALT (IU/L)	13.01	12.04	10.40	12.96	0.741	0.049	0.134	0.782	0.015
BUN (mg/dL)	10.46	12.56	11.57	11.57	0.626	0.126	0.804	0.733	0.025
Ca (mg/dL)	6.48	6.54	5.98	6.52	0.125	0.024	0.431	0.291	0.005
IP (mg/dL)	5.81^b^	6.47^a^	5.81^b^	6.26^ab^	0.128	0.036	0.032	0.480	0.032
Mg (mg/dL)	1.37	1.35	1.22	1.34	0.045	0.392	0.451	0.387	0.202
Cholesterol (mg/dL)	76.87	104.57	68.98	77.21	10.634	<0.001	0.008	0.924	<0.001
Triglycerides (mg/dL)	7.01	7.71	9.76	8.98	0.869	0.017	0.922	0.130	0.004
Glucose (mg/dL)	69.71	74.75	66.50	73.89	2.083	0.138	0.130	0.746	0.069
Albumin (IU/L)	1.93	2.03	1.80	1.93	0.050	0.073	0.232	0.247	0.029
Creatinine (mg/dL)	0.82^a^	0.70^b^	0.80^a^	0.73^ab^	0.021	0.009	0.269	0.002	0.313
NEFA (mEq/L)	0.15	0.17	0.14	0.14	0.027	0.787	0.708	0.877	0.373

SEM, standard error of the mean; AST, aspartate transaminase; ALT, alanine transaminase; BUN, blood urea nitrogen; IP, inorganic phosphate; NEFA, non-esterified fatty acid.

1) HS, high starch concentrate; HF, high fiber concentrate.

a,b Means that do not have common superscripts significantly differ within the treatments (p<0.05).
